# Impacts of DNA ligase I genotypes on Taiwan Parkinson’s disease

**DOI:** 10.37796/2211-8039.1681

**Published:** 2026-06-01

**Authors:** Chao-Hsuan Chen, Wen-Shin Chang, Chia-Wen Tsai, Da-Tian Bau, Shih-Wei Hsu, Kun-Ting Hong, Chon-Haw Tsai, Fuu-Jen Tsai, Ming-Kuei Lu, Jai-Sing Yang

**Affiliations:** a Department of Neurosurgery, China Medical University Hospital, Taichung, Taiwan; b Graduate Institute of Biomedical Sciences, China Medical University, Taichung, Taiwan; c Terry Fox Cancer Research Laboratory, Department of Medical Research, China Medical University Hospital, Taichung, Taiwan; d Office of Research and Development, Asia University, Taichung, Taiwan; e Division of Neurosurgery, Department of Surgery, Taichung Armed Forces General Hospital, Taichung, Taiwan; f National Defense Medical University, Taipei, Taiwan; g Department of Medical Research, China Medical University Hospital, China Medical University, Taichung, Taiwan; h School of Chinese Medicine, College of Chinese Medicine, China Medical University, Taichung, Taiwan; i China Medical University Children’s Hospital, Taichung, Taiwan; j Department of Medical Genetics, China Medical University Hospital, Taichung, Taiwan; k Division of Parkinson’s Disease and Movement Disorders, Department of Neurology, China Medical University Hospital, Taichung, Taiwan

**Keywords:** DNA ligase I, Genotypes, Parkinson’s disease (PD), Single nucleotide polymorphism (SNP), Taiwanese Han population

## Abstract

**Introduction:**

Genetic polymorphisms in DNA repair pathways can modulate DNA repair capacity and influence susceptibility to aging-related disorders, including Parkinson’s disease (PD). This study evaluated the association between the DNA ligase I rs20579 polymorphism and PD risk.

**Materials and methods:**

Genotyping of DNA ligase I rs20579 was performed by polymerase chain reaction-restriction fragment length polymorphism (PCR-RFLP) in 123 PD patients and 492 age- and sex-matched controls of Taiwanese Han ethnicity. Associations between rs20579 genotypes and PD risk were analyzed.

**Results:**

Among controls, the genotype distribution of rs20579 was 78.7 % GG, 19.3 % AG, and 2.0 % AA, compared with 75.6 %, 21.1 %, and 3.3 % among PD cases, respectively (p for trend = 0.6290). No significant differences were observed in the prevalence of AG (p = 0.6939) or AA (p = 0.4908) genotypes between cases and controls. Allelic analysis showed a non-significant increase in PD risk for A allele carriers (OR = 1.21, 95 % CI = 0.80–1.83, p = 0.4189). Stratified analysis revealed no significant genotype differences among males. In females, the AA genotype was associated with a borderline increased risk of PD (OR = 1.86, 95 % CI = 1.00–3.47, p = 0.0526).

**Conclusion:**

In this Taiwanese Han cohort, the DNA ligase I rs20579 A allele may be a genetic marker of increased PD susceptibility in females. Further studies in larger, independent populations are warranted to confirm its clinical relevance.

## 1. Introduction

Parkinson’s disease (PD) is a progressive neurodegenerative disorder whose prevalence increases markedly with advancing age [1,2]. Clinically, PD is characterized by cardinal motor symptoms, including resting tremor, muscular rigidity, bradykinesia, and impaired postural reflexes [3,4]. As a leading cause of morbidity, PD imposes substantial healthcare costs and socioeconomic burdens [5,6]. Despite extensive research, the mechanisms underlying PD pathogenesis and its clinical heterogeneity remain incompletely understood [3,7]. Accordingly, identifying sociodemographic, genetic, and environmental determinants that contribute to PD susceptibility, progression, or prevention remains a critical research priority.

Aging is the most significant risk factor for PD; however, the mechanisms by which age-related processes contribute to PD pathophysiology remain incompletely understood [8]. At the apex of biological information storage, DNA is uniquely vulnerable to damage, and its modification can lead to persistent, transmissible, and deleterious consequences in Parkinsonism [8,9]. Experimental evidence from mutant mouse models and human studies demonstrates that defects in specific DNA repair pathways accelerate systemic aging and promote progeroid syndromes, recapitulating many hallmarks of natural aging but with markedly faster progression [10]. DNA ligase I plays a central role in maintaining genome stability by catalyzing the sealing of single-strand breaks that occur during DNA replication and repair [10,11]. Amino acid substitutions within the proliferating cell nuclear antigen (PCNA)-interacting protein have been shown to disrupt PCNA binding, hinder the nuclear localization of DNA ligase I to the exact sites of replication, and impair the ligation of Okazaki fragments [12,13]. The flexible and intrinsically disordered N-terminal domain of DNA ligase I additionally regulates its interactions with replication-associated proteins, including Rad9-Rad1-Hus1 and replication factor C (RFC) [14,15]. This non-catalytic region also facilitates interactions with polymerase β [16], underscoring its multifaceted role in replication and repair.

**Fig. 1:**
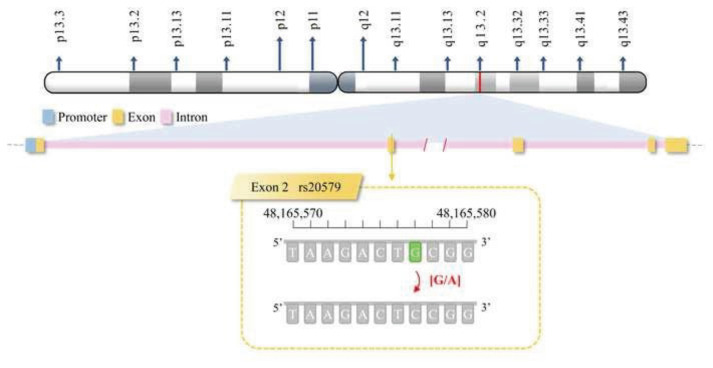
Physical map of the DNA ligase I rs20579 polymorphic site.

Genetic studies have linked DNA ligase I polymorphisms to cancer risk. Chang and colleagues reported that although individual DNA ligase I variants were not associated with lung cancer risk, a GGGAA haplotype comprising rs20581-rs156641-rs3730931-rs20579-rs439132 SNPs conferred a reduced risk of disease [17]. The *DNA ligase I* rs20579 polymorphism, an intronic variant merged from rs117149496, rs17402855, rs16981751, and rs3730848, has not yet been functionally characterized in terms of its impact on DNA ligase I mRNA or protein expression [18,19]. Nevertheless, it has been hypothesized that this subtle variant may influence overall DNA repair capacity. Polymorphisms in DNA ligase I, including rs20579, have been implicated in susceptibility to several cancers, including lung cancer [19], endometrial cancer [20], leukemia [21] and glioma [22]. Given the central role of DNA ligase I in genome stability, it is plausible that rs20579 variant genotypes may impair DNA repair efficiency, contributing to PD initiation and progression Fig.1.

To date, no study has investigated the potential influence of DNA ligase I rs20579 polymorphic alterations on the risk of developing PD. In the present work, we conducted a comprehensive study in a representative Taiwanese Han population (cases: controls = 123: 492), marking the first investigation worldwide to evaluate the contribution of DNA ligase I rs20579 to PD susceptibility.

## 2. Materials and methods

### 2.1. Recruitment of PD cases and age- and gender-matched controls

All patients with PD were diagnosed by board-certified neurologists. Each participant completed a personal questionnaire and provided an individual peripheral blood sample. Healthy controls were randomly and blindly selected and matched to cases for age (±4 years) and gender at a 4:1 ratio. All study participants were of genetically conserved Taiwanese descent. The study protocols were approved and supervised by the Institutional Review Board of China Medical University Hospital (IRB: CMUH109-REC2-133) and conducted in accordance with the Declaration of Helsinki. The demographic characteristics of the PD and non-PD populations, including age and gender distributions, are shown in Table 1.

### 2.2. DNA ligase I genotyping methodology among PD cases and age-and gender-matched controls

Genomic DNA was isolated within 24 h after collection using previously published protocols [23,24]. DNA quantity and quality were assessed, and samples were stored long-term at −80 °C, while aliquots were maintained at −20 °C as working stock. The genotyping assay for *DNA ligase I* rs20579 was developed at the Terry Fox Cancer Research Laboratory, China Medical University Hospital, as previously described [21]. The primers used were 5′-AATCTCCAGACGCTGCCAGA-3′ (forward) and 5′-CTCTGCACAACCAATCACCT-3′ (reverse), respectively. Polymerase chain reaction (PCR) process was conducted with the following settings: an initial denaturation at 94 °C for 5 min; 35 cycles of 30 s at 94 °C, 30 s at 52 °C, and 30 s at 72 °C; followed by a final extension at 72 °C for 10 min. PCR products were digested with *BsaH* I restriction enzyme and resolved on 3 % agarose gels at 100 V for 25 min. Genotypes were determined by banding patterns: AA produced a single 402-bp fragment; AG yielded three fragments (402, 303, and 99 bp); and GG generated two fragments (303 and 99 bp). All genotyping was conducted in a double-blind fashion, with each sample independently analyzed by at least two researchers (co-authors or acknowledged contributors). Notably, the genotyping success rate was 100 %, with throughout concordance across all results [25].

### 2.3. Statistical analysis

The fitness of Hardy–Weinberg equilibrium (HWE) for *DNA ligase I* rs20579 genotypes among the collected 492 non-PD controls was assessed using the goodness-of-fit chi-square test. Differences in mean age between PD cases and controls were evaluated using an unpaired *Student’s t*-test. Genotype distributions within subgroups were compared using Pearson’s chi-square test with Yates’ correction, or Fisher’s exact test when expected cell counts were <5. Logistic regression analysis was applied to estimate odds ratios (ORs) and 95 % confidence intervals (CIs), thereby assessing the association between rs20579 genotypes and PD risk. Any P*-*value <0.05 (two-tailed) was considered statistically significant [26].

## 3. Results

The distributions of demographic variables for the 123 PD cases and 492 controls are summarized in Table 1. The two counterpart groups were well matched for both age and gender, with no any significant difference detected between PD and non-PD groups ( *p* = 0.6855 and 1.0000, respectively). When participants were stratified by age (<69 years vs. ≥69 years), no significant differences were detected between PD case and non-PD control groups ( *p* = 0.9839).

The distribution pattern of *DNA ligase I* rs20579 polymorphisms among the 492 non-PD controls and 123 PD cases is shown in Table 2. The genotypic frequencies in the control group conformed to HWE ( *p* = 0.1519). The overall distribution of rs20579 genotypes did not differ significantly between PD cases and non-PD controls ( *p* for trend = 0.6290). Compared with the reference GG genotype, neither the heterozygous AG genotype (OR = 1.14, 95 % CI = 0.70–1.86, *p* = 0.6939) nor the homozygous AA genotype (OR = 1.66, 95 % CI = 0.51–5.42, *p* = 0.4908) was significantly associated with PD risk. In the recessive model (AA vs. GG + AG), individuals carrying the AA genotype exhibited a 1.62-fold increase in PD risk, though the association was not statistically significant (95 % CI = 0.50–5.26, *p* = 0.4953). Similarly, in the dominant model (AG + AA vs. GG), carriers of at least one A allele showed a non-significant 1.19-fold increase in risk (95 % CI = 0.75–1.89, *p* = 0.5426; Table 2).

To further corroborate the findings presented in Table 2, we conducted an allelic frequency analysis of *DNA ligase I* rs20579, with results shown in Table 3. This analysis reinforced the conclusion that the *DNA ligase I* rs20579 polymorphism is not associated with PD susceptibility. Specifically, the frequency of the variant A allele was 13.8 % in PD cases and 11.7 % in non-PD controls (OR = 1.21, 95 % CI = 0.80–1.83, *p* = 0.4189). These results indicate no statistically significant difference in allelic distribution between the PD and non-PD groups (Table 3).

We further evaluated the combinative effects of *DNA ligase I* rs20579 genotypes and gender on PD susceptibility. Among males, neither the heterozygous AG genotype (OR = 0.86, 95%CI = 0.44–1.66, *p* = 0.7684) nor the homozygous AA genotype (OR = 0.97, 95 % CI = 0.20–4.69, *p* = 1.0000) was associated with a significant change in PD risk. Allelic frequency analysis in males yielded similar non-significant results (Table 4, upper section). In contrast, in females, the homozygous AA genotype showed a borderline increase in PD risk (OR = 4.73, 95 % CI = 0.64–34.95, *p* = 0.1516). Allelic frequency analysis revealed a similarly borderline significant association, with carriers of the A allele exhibiting an increased risk of PD (OR = 1.86, 95 % CI = 1.00–3.47, *p* = 0.0526; Table 4, lower section). These findings suggest a potential sex-specific effect of the rs20579 variant on PD susceptibility in females.

## 4. Discussion

Cells must maintain genomic integrity and proliferative capacity throughout aging, necessitating a nearly error-free DNA repair system. As mentioned in the Introduction, DNA ligase I is a key enzyme in both DNA replication processes and the repair of DNA strand breaks [8,9,27,28] In individuals with DNA ligase I deficiency syndrome, which manifests with immunodeficiency, Maffucci and his colleagues have identified several homozygous and/or heterozygous mutations on *DNA ligase I* as the underlying cause for the disease [28,29]. *In vitro* studies using DNA ligase I-null human 293 T cells have demonstrated a significant suppression of overall cell viability following DNA damage, which can be rescued by reinforcement of wild-type DNA ligase I [28]. Several inherited mutation sites in DNA ligase I, including P529L, R641L, R771W, and E566K, have been shown to cause DNA ligase I reduction and/or deficiency, which may result in functional immunodeficiency and/or severity [30,31]. Functional studies demonstrated that 293 T cells expressing the R641L or R771W variants showed a moderate decline in cell survival following treatment with the DNA-alkylating compound ethyl methanesulfonate, indicative of a partial defect in DNA repair capacity [30,31]. In agreement with these findings, murine models carrying the R771W mutation display elevated genomic instability and an increased propensity for tumor development [32,33], whereas patient-derived B and T lymphocytes with the R641L substitution exhibit diminished repair responses following ionizing radiation exposure [31,34]. Moreover, as early as 2001, aberrant over-expression of DNA ligase I was reported in several human solid tumors, implicating its dysregulation in oncogenic processes [21,28]. Collectively, these observations underscore that compromised DNA ligase I activity undermines genomic stability, thereby driving the development of multiple human pathologies, most notably cancer. Nonetheless, its potential involvement in PD remains unexplored.

In the current study, we examined the contribution of *DNA ligase I* rs20579 polymorphic genotypes to PD risk in a representative Taiwanese Han population. First, we determined the distribution of rs20579 genotypes among non-PD healthy controls and compared these frequencies with global population dataset available from the online and free National Center for Biotechnology Information (NCBI) databases. The dataset kept and updated the genotyping records of 528 individuals with Asian ancestry, subdivided into 424 East Asians and 104 other Asians, enabling a detailed evaluation of minor allele frequencies (MAF). The calculated MAFs at rs20579 were 0.1100 for all Asians, 0.1110 for East Asian and 0.1060 for other Asians (URL: https://www.ncbi.nlm.nih.gov/snp/rs20579). In comparison, the MAF in 101,222 Europeans was 0.1234, similar to the Asian populations, whereas substantially higher MAFs were observed in 14,018 Africans (0.2802) and 13,620 African Americans (0.2790), reflecting the marked genotypic divergence among these populations. In our Taiwanese Han population cohort, the minor allele A was detected at a frequency of 0.1380 (Table 3), closely aligning with the frequencies reported for both East Asian and other Asian populations.

Subsequently, we evaluated the association between *DNA ligase I* rs20579 polymorphisms and PD susceptibility within a genetically homogeneous and conserved central Taiwan population. Our cohort, comprising 123 PD cases and 492 non-PD controls, provides a highly representative sample, particularly when compared with a recently published study funded with substantial official resources [44], which included fewer controls (n = 250) and slightly more PD cases (n = 150). The detailed questionnaires reported in that study served as a valuable reference for our design [35]. Our results revealed no positive association between the AG or AA genotypes of *DNA ligase I* rs20579 and PD susceptibility (Tables 2 and 3).

The findings provide evidence suggesting that *DNA ligase I* rs20579 genotype is unlikely to have a major effect on DNA ligase I function, which, if impaired, could compromise DNA repair and increase PD risk, particularly in females (Table 4). This underscores the likelihood that other polymorphic loci may play a more critical role in modulating DNA ligase I activity and influencing PD susceptibility. Further studies are needed to identify additional genetic variants that may serve as predictive biomarkers for PD risk.

## 5. Conclusion

In summary, our findings indicate that the variant A allele of *DNA ligase I* rs20579 does not serve as a reliably practical biomarker for predicting personal susceptibility to PD in Taiwan. Future research should explore the effects of other polymorphic variants within the *DNA ligase I* gene and examine their potential genotype-phenotype associations with PD. Moreover, a comprehensive evaluation of how *DNA ligase I* genotypes interact with broader DNA repair pathways is warranted. Ultimately, in-depth mechanistic studies are needed to clarify the role of *DNA ligase I* in the pathogenesis of PD.

## Figures and Tables

**Table 1:** Distribution of basic demographic characteristics in 123 PD patients and 492 controls.

	Characteristics	Controls (n = 492)	PD patients (n = 123)	*p-*Value
Age, years	Mean ± SD	69.4 ± 6.8	69.1 ± 6.8	0.6855^a^
	<69 Years	263 (53.5 %)	65 (52.8 %)	
	≥69 Years	229 (46.5 %)	58 (47.2 %)	0.9839^b^
Gender, n (%)	Male	312 (63.4 %)	78 (63.4 %)	
	Female	180 (36.6 %)	45 (36.6 %)	1.0000^b^

PD: Parkinson disease; SD: Standard deviation.

a Based on unpaired *Student’s t*-test.

b Based on chi-square test with Yates’ correction.

**Table 2:** Distribution of DNA ligase I rs20579 genotypes among 123 PD patients and 492 controls.

Genotypes	Controls, N	%	PD cases, N	%	OR (95 % CI)	*p*-value
*Ligase I* rs20579						
GG	387	78.7	93	75.6	1.00 (Ref)	
AG	95	19.3	26	21.1	1.14 (0.70–1.86)	0.6939^a^
AA	10	2.0	4	3.3	1.66 (0.51–5.42)	0.4908^b^
*P*_trend_						0.6290
*P*_HWE_						0.1519
Carrier comparison						
GG + AG	482	98.0	119	96.7	1.00 (Ref)	
AA	10	2.0	4	3.3	1.62 (0.50–5.26)	0.4953^b^
GG	387	78.7	93	75.6	1.00 (Ref)	
AG + AA	105	21.3	30	24.4	1.19 (0.75–1.89)	0.5426^a^

N: Number; PD: Parkinson disease; OR: odds ratio; CI: Confidence interval; Ref: reference group.

a Based on Chi-square test with Yates’ correction.

b Based on Fisher’s exact test; *P*_trend_: *p*-value for trend analysis; *P*_HWE_: whether the frequencies in control subjects are consistent with Hardy–Weinberg Equilibrium.

**Table 3:** Distribution of DNA ligase I rs20579 allelic frequencies among 123 PD patients and 492 controls.

Allele	Controls, N	%	PD cases, N	%	OR (95 % CI)	*p-*valuea
*Ligase 1* rs20579						
G	869	88.3 %	212	86.2 %	1.00 (Reference)	
A	115	11.7 %	34	13.8 %	1.21 (0.80–1.83)	0.4189

N: Number; PD: Parkinson disease; OR: odds ratio; CI: Confidence interval.

a Based on Chi-square with Yates’ correction test.

**Table 4:** Distribution of DNA ligase I rs20579 genotypes and allelic frequencies stratified by gender among 123 PD patients and 492 controls.

Genotypes	Controls, N	%	PD cases, N	%	OR (95 % CI)	*p*-value^a^
*Males*						
GG	245	78.5	63	80.8	1.00 (Ref)	
AG	59	18.9	13	16.7	0.86 (0.44–1.66)	0.7684^a^
AA	8	2.6	2	2.5	0.97 (0.20–4.69)	1.0000^b^
Allele						
G	549	88.0	139	89.1	1.00 (Ref)	
A	75	12.0	17	10.9	0.90 (0.51–1.56)	0.8028^a^
*Females*						
GG	142	78.9	30	66.7	1.00 (Ref)	
AG	36	20.0	13	28.9	1.71 (0.81–3.61)	0.2250^a^
AA	2	1.1	2	4.4	4.73 (0.64–34.95)	0.1516^b^
Allele						
G	320	88.9	73	81.1	1.00 (Ref)	
A	40	11.1	17	18.9	1.86 (1.00–3.47)	0.0526^a^

N: Number; PD: Parkinson disease; OR: odds ratio; CI: Confidence interval; Ref: reference group.

a Based on Chi-square test with Yates’ correction.

b Based on Fisher’s exact test; *P*_trend_: *p*-value for trend analysis.
